# Environmental exposure assessment framework for nanoparticles in solid waste

**DOI:** 10.1007/s11051-014-2394-2

**Published:** 2014-05-14

**Authors:** Alessio Boldrin, Steffen Foss Hansen, Anders Baun, Nanna Isabella Bloch Hartmann, Thomas Fruergaard Astrup

**Affiliations:** Department of Environmental Engineering, Technical University of Denmark, Miljoevej, 2800 Kongens Lyngby, Denmark

**Keywords:** Nanowaste, Nanomaterial, Environmental exposure, Solid waste, Quantification, Health effects

## Abstract

Information related to the potential environmental exposure of engineered nanomaterials (ENMs) in the solid waste management phase is extremely scarce. In this paper, we define nanowaste as separately collected or collectable waste materials which are or contain ENMs, and we present a five-step framework for the systematic assessment of ENM exposure during nanowaste management. The framework includes deriving EOL nanoproducts and evaluating the physicochemical properties of the nanostructure, matrix properties and nanowaste treatment processes as well as transformation processes and environment releases, eventually leading to a final assessment of potential ENM exposure. The proposed framework was applied to three selected nanoproducts: nanosilver polyester textile, nanoTiO_2_ sunscreen lotion and carbon nanotube tennis racquets. We found that the potential global environmental exposure of ENMs associated with these three products was an estimated 0.5–143 Mg/year, which can also be characterised qualitatively as medium, medium, low, respectively. Specific challenges remain and should be subject to further research: (1) analytical techniques for the characterisation of nanowaste and its transformation during waste treatment processes, (2) mechanisms for the release of ENMs, (3) the quantification of nanowaste amounts at the regional scale, (4) a definition of acceptable limit values for exposure to ENMs from nanowaste and (5) the reporting of nanowaste generation data.

**Electronic supplementary material:**

The online version of this article (doi:10.1007/s11051-014-2394-2) contains supplementary material, which is available to authorized users.

## Introduction

The production of engineered nanomaterials has been increasing steadily over the past decade. Engineered nanomaterials (ENMs) (of which engineered nanoparticles are considered a subset, see Hansen et al. 2007) are used today in a wide range of nanoproducts (defined as finished goods containing ENMs) and applications worldwide (PEN 2009; The Nanodatabase 2013). Since 2005, the production of nanoproducts has increased by approximately 250 new nanoproducts every year (PEN 2009). Over time, the manufacturing and use of nanoproducts will result in the generation of increasing amounts of waste containing nanomaterials (Dang et al. 2010) as well as waste from the production processes themselves. Despite the fact that most of the nanoproducts and affected waste can be expected to eventually end up in regular waste streams, for example, as part of the municipal solid waste management system, very little is known about the quantities of waste (Reinhart et al. 2010; Health Council of the Netherlands 2011), while the consequences of ENMs entering waste streams are yet unclear (Health Council of the Netherlands 2011; Arvidsson et al. 2012). Due to the large variety of nanoproducts, the toxicity potential of nanomaterials and the wide range of potentially affected waste streams, the consequences for future waste management are currently unpredictable. Improved understanding of the flows and fates of ENMs within the waste management system is therefore required (Health Council of the Netherlands 2011).

Upto this point, significant attention has been paid to establishing the inherent hazardous properties of ENMs. The environmental exposure of ENMs found in waste, however, has received less attention, and next to nothing is known about the overall risks of nanoparticles in solid waste materials (Arvidsson et al. 2012). Solid waste containing ENMs may not be identified as such, though, and currently waste is not managed separately but is rather collected and treated together with ‘regular’ waste. ENM release into the environment may take place during all steps in a waste management system (e.g. collection, recycling, incineration and landfilling). During waste *collection*, ENMs may be released due to abrasion as a result of waste compaction and handling (Roes et al. 2012). For *recycling*, Köhler et al. (2008) reported that carbon nanotubes (CNTs) may be released and emitted during shredding, milling, sorting and thermal processing, resulting in possible direct exposure in the working environment. Next, the thermal properties of ENMs may determine their fate during the *incineration* of nanoproducts; for example, Cataldo (2002) demonstrated that C_60_ molecules were less stable thermally than CNTs, because during combustion, C_60_ behaves like graphite, while CNTs, similar to diamonds, are stable, until they reach very high temperatures. The subsequent release of ENMs mixed with flue gas from the incineration of waste containing ENMs may be affected not only by the thermal stability of the ‘host’ or ‘matrix’ material but also by the flue gas cleaning system. The few available studies which address the incineration of nanoproducts have indicated that ENM removal efficiencies may vary significantly and depend on properties such as particle type and size (Huang and Chen 2002). For example, while removal efficiencies in wet scrubbers (Walser et al. 2011) and electrostatic precipitators (Bologa et al. 2009) are reported to be close to 100 % for 80 nm nanoCeO_2_ and ultrafine particles at 100 nm, other studies have indicated a removal efficiency lower than 50 % for ENMs smaller than 50 nm (Li et al. 2009). Overall, the mechanisms controlling ENMs emissions from the thermal treatment of waste remain to be studied in detail. Today, *landfilling* is the most widely applied waste management option (e.g. 36 % in EU-27 in 2011, as reported in Eurostat 2012). While more than 50 % of ENMs produced worldwide may be landfilled (Reinhart et al. 2010; Keller et al. 2013), their long-term behaviour in landfills is still largely unknown (Asmatulu et al. 2012). ENM emissions from landfills most likely depend on the properties of the ENMs, the waste materials containing the ENMs and the physicochemical and hydrological conditions in the landfill body. ENM mobility in landfills is, therefore, affected by a range of variables, and the final release into the environment is poorly described (Nowack et al. 2012).

Existing waste regulations do not contain specific references to ENMs, although ENMs have been addressed explicitly in other recently adopted regulations (e.g. the European Cosmetics Regulation and Biocidal Products Regulation). As end-of-life (EOL) nanoproducts may not be readily identifiable as nanoproducts, it can be assumed that nanoproducts and waste containing nanomaterials are not managed as a specific waste stream. Consequently, the fate of ENMs in waste is determined by the properties of the waste material or product containing them, which means that nanoproducts in some cases may fall into a specific waste category, for example, ‘oil lubricants containing C_60_,’ as existing regulations require the specific treatment of spent lubricant (Franco et al. 2007). In other cases, ENMs will be regulated as part of the ‘generic’ solid waste stream for mixed municipal solid waste (e.g. the Waste Framework (Directive 2006/12/EC) in the EU and the RCRA act in the USA). In yet other cases, waste containing nanoproducts or ENMs may be regulated as hazardous waste (e.g. European Commission 2000) in the EU, the RCRA Act in the USA (Beaudrie et al. 2013) or as waste electrical and electronic equipment (Directive 2012/19/EU). As ENMs are not mentioned explicitly, it is yet unclear as to what extent nanoproducts may be considered hazardous and thereby be affected by the specific requirements of hazardous waste management policy.

Current waste-related regulations do not contain specific requirements to address properly the presence of nanoproducts and waste containing ENMs (Health Council of the Netherlands 2011). So far, risk assessments have focused mainly on emissions during production and use, while little attention has been paid to the waste management phase, on reason for which is that waste is not considered a chemical substance, and hence most obligations under REACH do not apply to waste. Under REACH, chemical manufacturers and/or importers have to document that risks associated with a given chemical substance can be managed properly in the waste lifecycle. In practice, this means that an exposure assessment (including an environmental exposure assessment) has to be carried out for the waste life stage when (1) the substance subject to registration under REACH is produced or imported in quantities of 10 Mg or more per year, per registrant, and (2) the substance meets criteria for classification as dangerous according to Regulation (EC) No 1272/2008. Based on the exposure assessment, the safety data sheet of a substance should then (1) identify a proper waste management approach for the substance or mixture and/or its container, (2) specify physical and chemical properties that may affect waste treatment options and (3) describe special precautions required for any recommended waste treatment option. However, without better and more detailed data on the flows and fate of ENMs within the waste management system, specific requirements for nanowaste cannot be identified, which could potentially lead to uncontrolled exposure in the environment.

This paper aims at providing an improved basis for decision-making in relation to the waste management of nanoproducts and waste containing ENMs. This will be achieved by providing: (1) a definition of ‘nanowaste’, acknowledging the constraints of existing waste management, (2) a five-step environmental exposure assessment framework for ENMs in solid waste, (3) quantification of the amounts and fates of three selected nanoproducts in waste streams and (4) identification of critical challenges in relation to the characterisation of waste in view of ENMs and the associated reporting of waste containing ENMs. The intention is to facilitate informed decision-making when establishing the waste-specific regulation of nanomaterials, as well as to direct future research towards critical aspects of waste management.

## A definition of nanowaste

Nanomaterials have been defined as materials with a structure of 1–100 nm in at least one dimension, where the nanostructure provides specific properties, thus making ENMs different from their corresponding bulky system (Nanoscale Science Engineering and Technology Subcommittee 2004). In 2011, the European Commission adopted a Recommendation on the definition of a nanomaterial (EC 2011), generally not only referring to materials containing particles for which 50 % or more have external dimensions of 1–100 nm, but also including fullerenes, graphene flakes and single-wall CNTs, even if they have one or more dimensions below 1 nm. In our definition of nanowaste, as set out below, we apply this definition of ENMs.

Musee (2011a) proposed a definition of nanowaste as ‘waste stream(s) containing ENMs, or synthetic by-products of nanoscale dimensions, generated either during production, storage and distribution, or waste stream(s) resulting from the end of a lifespan of formerly nanotechnologically enabled materials and products, or items contaminated by ENMs such as pipes, personal protection equipment, etc’.. Under the premise of nanomaterials being used in a wide range of applications and consumer products, and that the resulting nanoproducts may not necessarily be identifiable by consumers at their EOL stage, this definition has two problems: (1) a waste stream, e.g. household waste, is defined as ‘nanowaste’ simply, because small quantities of nanomaterials are present in certain EOL products and (2) waste materials which could be collected and separately treated are not distinguished from waste contaminated (perhaps unintentionally) with nanomaterials, thereby not facilitating separate collection. As such, no distinction between collectable and non-collectable nanowaste is provided in the definition given by Musee (2011a), and potentially, all waste flows in society could fall under the definition of ‘nanowaste’.

**Fig. 1:**
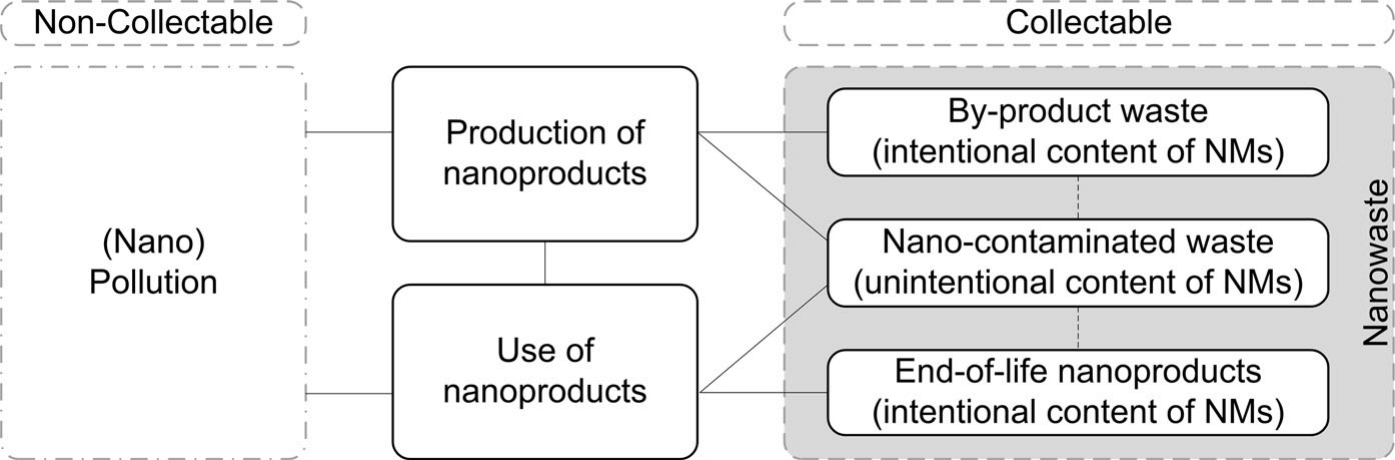
Generation of solid waste containing ENMs (nanowaste) throughout the lifecycle of nanoproducts. Nanowaste is shaded in *gray*. Nano-contaminated waste can originate from both the production and use phases of nanoproducts, and occasionally from waste treatment

It must be a minimum requirement for the practical usability of a nanowaste definition with waste management that it reflects the characteristics of the waste management system and provides guidance with respect to the management of ENM-containing waste. We propose to limit the scope of the definition of nanowaste to include only separately collected or collectable waste materials *which are or contain* ENMs. This means that nanowaste can include (1) ENMs as a single fraction, e.g. by-products from manufacturing of nanoproducts, (2) EOL nanoproducts and (3) individual waste materials contaminated with ENMs, for example, sludge from wastewater treatment. It should be noted that the fact that nanowaste shall be either collected separately or collectable distinguishes it from pollution (definitions of ‘waste’ and ‘pollution’ are provided in the supporting information). Within this definition, ENMs emitted directly into the environment are not considered nanowaste and should rather be considered as ‘nanopollution’, examples of which include nanosilver washed from T-shirts as well as nanomaterials in cosmetics that enter into wastewater streams after washing, showering, etc. See Fig. 1 for an overview.

A consequence of the above definition is that waste which cannot or is not collected separately (either due to practical limitations of the waste management system or because of requirements related to the matrix materials ‘hosting’ the ENMs) is not considered as nanowaste. This means that ENMs may be present in waste streams without the waste being characterised as nanowaste and without these ENMs inducing a need for special and separate management of the waste. Therefore, nanomaterials in waste are considered a contaminant, e.g. similar to polychlorinated biphenyls (PCBs), dioxins or mercury, and not a property of the waste itself. It is only if the ENM contamination level becomes problematic, and special treatment is required that the waste should be collected separately as nanowaste. This could be regulated, for example, in a similar fashion to what is currently done for hazardous waste in existing legislation in the EU and the USA (e.g. Council Directive 1991/689; EC 2000; US Government 2012). A quantitative risk-based definition of threshold values, determined for individual ENMs and specified for different hazardous properties (e.g. toxicity, carcinogenesis; see supporting information for a complete list of properties), however, will only be possible when substantial information about these properties is made available.

## Procedure for the environmental exposure assessment of nanomaterials in solid waste

**Fig. 2:**
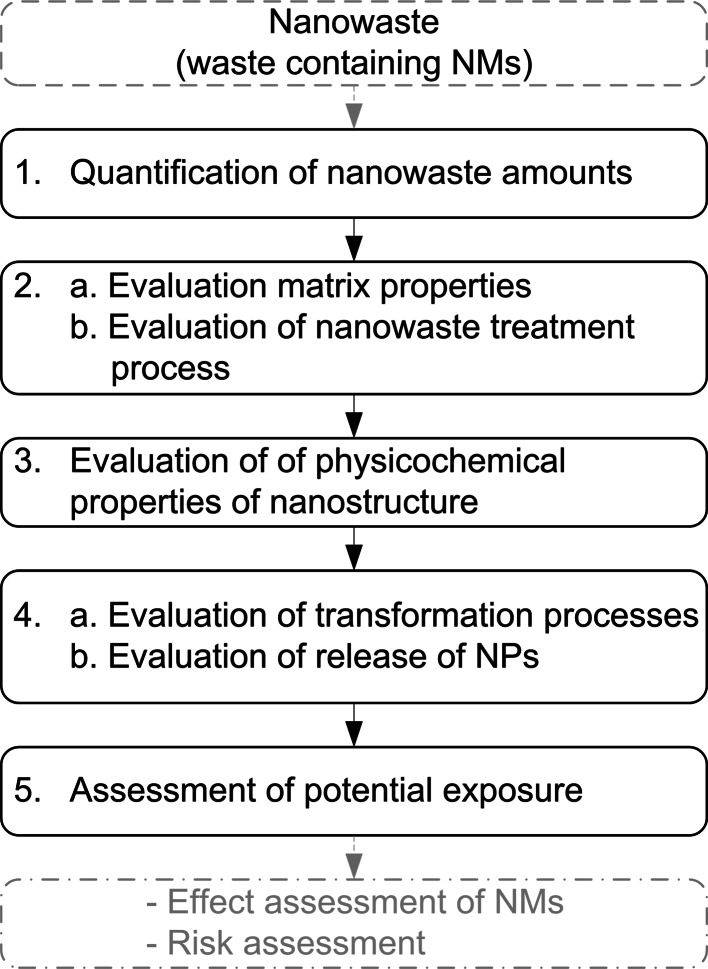
Proposed framework for an environmental exposure assessment of nanoparticles in solid waste. The framework includes steps 1–5. When combined with results from an effect assessment, the results of the exposure assessment may be used as an input into the environmental risk assessment of nanoparticle emissions from waste (*lower dotted box*, outside the scope of the present study)

The paradigm for assessment of risk, exposure and effects of chemicals was considered by Musee (2011b) as a starting point for the risk-based classification of nanowaste. However, due to a lack of both toxicity data and exposure information, this approach is currently not able to deliver the outputs required for decision-making. Focus may therefore be directed towards assessing environmental exposure related to nanowaste management, which requires knowledge about the handling, processing and disposal of nanowaste as well as related environmental emissions (Upadhyayula et al. 2012). In relation to waste management, a number of specific aspects may influence the emission of ENMs from nanowaste into the environment, for example, the physicochemical properties of both the waste matrix and the ENMs themselves, as well as any transformation processes that nanowaste undergoes during handling/treatment/disposal in the waste management system. In order to address systematically these complex interactions, we propose a stepwise assessment framework, as outlined in Fig. 2 and described in the following sections. The use of the framework is then illustrated using three nanoproduct examples. While the three examples focus on EOL nanoproducts, a similar approach can be employed to determine environmental exposure related to nanowaste arising from ENM manufacturing processes.

### Step 1: Quantification of nanowaste amounts

In this study, as also suggested by Keller et al. (2013), the quantification of nanowaste generation is done by market product analysis according to the lifecycle stage of the nanoproduct in question, i.e. (1) nanowaste generated as by-products from ENM manufacturing and (2) nanowaste as a result of EOL nanoproducts.

The market product analysis seems at present to be the only feasible way to make these kinds of quantifications, since data regarding nanowaste still do not exist, and appropriate analytical techniques for quantifying ENMs in waste materials need significant development in order to be of any practical value (see discussion in Sect. Characterisation of nanowaste – issues with analytical methods).

#### By-products from ENM manufacturing

Waste might be generated during ENM manufacturing, and this may or may not contain ENMs, whereby the latter scenario may be handled according to the local waste management system. Nanowaste may occur as a by-product of the manufacturing process as (1) rejected material from the ENM size selection stage (Köhler et al. 2008), (2) residual ancillary materials used for the manufacturing and/or purification of ENMs and (3) leftover surplus of raw material. Nanomaterial manufacturing can be considered a ‘low-yield’ process, with the majority of raw materials not ending up in the final product. Top-down manufacturing processes, for instance the production of ENMs by grinding larger sized materials, tend to generate more waste than bottom-up approaches such as ENM production through chemical synthesis (Dahl et al. 2007).

Nanowaste generated as a by-product of ENM manufacturing can be measured—in principle—by the producer or estimated assuming generation rates (i.e. a certain amount of waste per certain amount of ENM). By combining these generation rates with the amount of nanoproducts (or ENMs) produced, the amount of nanowaste generated as a by-product of manufacturing can be estimated. Data availability, however, is a critical aspect, and very little is currently available. Table 1 reports generation rates found in the existing literature for waste arising during the manufacturing of a range of ENMs. The data indicate that the amounts of waste generated from the manufacturing processes are in several cases significantly larger than the amount of the final ENM product. As mentioned above, this may be at least partly a consequence of the ‘low-yield’ processes involved. While not all waste reported in Table 1 is necessarily nanowaste according to our proposed definition, it is, however, plausible that a large share thereof contains ENMs to some extent, as the materials have been in direct contact with ENMs. A precise quantification may only be possible based on waste composition analyses.

**Table 1:** Examples of potential generation of nanowaste during manufacturing of nanoproducts

Waste	Generation rates		ENM type	Source	Comment
	Unit	Amount			
Ag^+^ in H_2_O solution	g g_product_^−1^	0.43	Ag	Tolaymat et al. (2010)	Probably discharged as wastewater
Trimethyl Aluminium [Al_2_(CH_3_)_6_]	g g_product_^−1^	0.98	Al_2_O_3_	Yuan and Dornfeld (2008)	Atomic layer deposition (ALD) process
Thiol solvent	L g_product_^−1^	15	Au	Dahl et al. (2007)	Purification process
Carbon soot	g g_product_^−1^	2–9	CNF	Khanna et al. (2007)	Vapour grown carbon nanofibers (VGCNFs)
Carbon soot	g g_product_^−1^	2–33	CNT-CNF	Zhang et al. (2011)	Review of various synthesis methods
Carbon soot	g g_product_^−1^	0.9	Fullerene	Royal Commission on Environmental Pollution (2008)	Sent to landfill
Carbon soot	g g_product_^−1^	7.22–25.6	Fullerene	Anctil et al. (2011)	Production: pyrolysis (input: toluene, tetralin), plasma RF/Arc (input: graphite)
Carbon soot	g g_product_^−1^	9	SWCNT	Seager et al. (2008)	SWCNT synthesis
Carbon soot	g g_product_^−1^	21.2	SWCNT	Isaacs et al. (2009)	Arc ablation (ARC) synthesis
Carbon soot	g g_product_^−1^	31.9	SWCNT	Isaacs et al. (2009)	Chemical vapour deposition (CVD) synthesis
Carbon soot	g g_product_^−1^	1250	SWCNT	Isaacs et al. (2009)	High pressure carbon monoxide (HiPco) synthesis
PTFE scrap membrane	g g_product_^−1^	11.91	SWCNT	Healy et al. (2008)	Purification after ARC synthesis
PTFE scrap membrane	g g_product_^−1^	6.17	SWCNT	Healy et al. (2008)	Purification after CVD synthesis
PTFE scrap membrane	g g_product_^−1^	5.73	SWCNT	Healy et al. (2008)	Purification after HiPco synthesis
Mix of ilmenite, iron powder, HCl	g g_product_^−1^	1.33	TiO_2_	Grubb and Bakshi (2011)	Altair(nano) hydrochloride process

*SWCNT* single-wall carbon nanotube, *CNF* carbon nanofiber

The data in Table 1 are somewhat in contrast with the previous literature. For example, Musee (2011b) stated that ‘during the production phase, nanowaste generation is most unlikely because closed reactors are used under vacuum conditions’, while Griffiths et al. (2013) excluded carbon soot by-products from the LCA modeling of CNT production. Although general conclusions cannot be drawn, due to limited data availability, this nevertheless indicates that the handling of nanowaste streams from ENM manufacturing should be considered a priority (also as pointed out by Chen et al. 2007 and Theis et al. 2011), as very little is known about how they should be controlled.

#### EOL nanoproducts

The amount of nanowaste generated from EOL nanoproducts depends on three critical factors, namely (1) the amount of nanoproducts produced and traded on the market (limited data are available), (2) the lifespan of ENMs or products containing them (which is difficult to predict, as this depends on consumer behaviour, material properties, etc.) and (3) the fraction of the virgin product reaching the EOL stage (i.e. loss of ENMs during the use phase). This fraction could depend on different factors such as the nature of the ENMs and/or the matrix carrying them. On this basis, the amount of nanowaste *X*_*t,p*_ [Mg year^−1^] generated in year *t* for nanoproduct type *p* (e.g. nanosilver-containing T-shirts) can be calculated as follows:

1 $$X_{t,p} = x_{t - rt,p} \times F_{{{\text{pen}},p}} \times F_{{{\text{eol}},p}}$$

where *x*_*t*-*rt,p*_ [Mg year^−1^] is the amount for product *p* produced in year *t* − *rt,* and *rt* is the retention time (i.e. duration of the use phase) of the product in the market.

*F*_pen*,p*_ [0→1]: a market penetration factor of nanoproduct *p*.

*F*_eol*,p*_ [0→1]: an EOL factor of nanoproduct *p*. This corresponds to the fraction of the virgin nanoproduct *p* reaching the EOL phase and thus becoming nanowaste.

Based on the amount of nanowaste *X*_*t,p*_ and information regarding the content of ENMs, the amount of nanomaterials *NM*_*t,p*_ [kg year^−1^] contained in nanowaste originating from nanoproduct type *p* in year *t* (e.g. nanosilver in textiles) can be calculated as shown in Eq. 2:

2 $$NM_{t,p} = X_{t,p} \times C_{{{\text{NM}},p}} \times F_{{{\text{NM}},p}}$$

with *X*_*t,p*_ [Mg year^−1^]: the amount of nanowaste in year *t* for nanoproduct type *p* (e.g. textiles containing nanosilver).

*C*_NM*,p*_ [mg Mg^−1^]: content of ENMs in nanoproduct *p.*

*F*_NM*,p*_ [0→1]: EOL factor for ENMs in nanoproduct *p*. This corresponds to the fraction of ENMs contained in nanoproduct *p* reaching the EOL phase and thus still contained in nanowaste. This accounts for the fact that a portion of ENMs contained in nanoproducts may be lost during the user phase.

### Step 2: Evaluation of matrix properties and nanowaste treatment processes

Nanowaste generated as a by-product can be assumed controllable, as the point of generation is identifiable, but this is not the case for EOL nanoproducts, which are expected to be present in regular waste flows. Moreover, as the presence of ENMs is not quantified in solid waste, EOL nanowaste management must be based on the properties of the matrix materials and/or the properties of the nanoproduct itself. The physical and chemical stability of the matrix material at conditions relevant for the type of waste treatment in question may further determine the release of ENMs into the environment. The physical and chemical properties of the waste matrix thereby become very important and should be decisive for the management of ENM-containing waste. The amount of ENMs entering individual waste material fractions varies significantly, depending on the specific nanoproduct in question (Köhler et al. 2008). The examples of EOL nanoproducts and affected solid waste types or individual material fractions presented in Table 2 show that a wide range of waste types and related treatment technologies may likely be affected by the presence of nanowaste. How individual waste material fractions are handled, treated and/or disposed depends on the local waste management system. Table 2 includes two examples of organic waste which may undergo biological treatment, during which part of the ENMs contained in the waste may be transferred to biosolids later applied to land (Lombi et al. 2012). Other examples are food waste containing NanoZnO, used as a food additive (Blasco and Picó 2011), or construction and demolition (C&D) waste containing paint with ENMs such as TiO_2_.

**Table 2:** Examples of EOL nanowaste and potential waste management affected

Nanoproduct	ENM	Matrix material	Matrix state	Nanostructure	Solid waste type or fraction	Waste management technology
Nanosilver textile	Ag	Cotton textile	Solid	Surface binding	Textiles	RE, RC, IN, LF
NanoTiO_2_ sunscreen	TiO_2_	Lotion cream Plastic flacon	Liquid	Suspension in liquid Surface binding (flacon)	Residual	IN, LF
CNT tennis racquet	CNT	Carbon fibre	Solid	Suspension in solid	Residual or bulky waste	IN, LF
NanoZnO in food additives	ZnO	Organic matter	Solid	Suspended in solid	Organic waste	BT, IN, LF
NanoTiO2 wall paint	TiO_2_	Paint Paint	Liquid Solid	Suspension in liquid Suspension in solid	Construction & demolition (C&D) waste	RE, LF
Nano-coated glass	TiO_2_	Glass	Solid	Surface binding	Glass	RE
Li-ion batteries	CNT	Mix of organic carbonates and lithium salts	Solid	Nanostructured in the bulk (anode)	Batteries	RE, IN
Circuit printboard	Various	Metal, plastic	Solid	Surface binding, Suspended in solid Nanostructured in the bulk	Waste electrical and electronic equipment (WEEE)	RE
WWTP sludge with NanoZnO^a^	ZnO	Organic matter	Solid	Suspension in solid	WWTP sludge	BT, UOL, IN

*WWTP* wastewater treatment plant, *RE* reuse, *RC* recycling, *BT* biotreatment, *UOL* use on land, *IN* incineration; *LF* landfill

^**a**^Secondary waste stream, not an EOL nanowaste

### Step 3: Evaluation of the nanostructure’s physicochemical properties

In addition to the physical and chemical properties of the matrix material (see previous section), the properties of the ENMs themselves—and their localisation in the matrix material—are also important for assessing the potential release mechanisms of ENMs into the environment. Hansen et al. (2007) provided a framework for categorising nanoproducts based on the location of the nanoscale structure in the matrix material. Within the framework, ENMs were divided into three main categories, depending on their presence: (1) in the ‘bulk’ of the material, (2) on the surface of the material and (3) as ‘free’ or aggregated nanoparticles. This categorisation relates mainly to EOL nanoproducts, but it may also be applicable to waste materials contaminated with nanomaterials (in these cases, attachment to the surface of materials is more likely). Based on Hansen et al. (2007), we have identified the following ENMs as being most prone to release:

- Surface nanofilms
- ENMs bound to the surface of another solid structure
- ENMs suspended in a liquid
- ENMs suspended in solids
- Airborne ENMs (in enclosed containers).

The extent to which they can be released into the environment therefore depends not only on the properties of the ENM, but also on the application in the nanoproduct and the general properties thereof (or nanowaste in general).

### Step 4: Evaluation of transformation processes and release of ENMs into the environment

The release of ENMs from nanowaste into the environmental compartments air, water and soil depends to a large extent on the specific conditions in the treatment technology (e.g. thermal vs. biological processes) and the potential transformations of the matrix materials and the ENMs themselves. Potential processes affecting ENMs were reported by Nowack et al. (2012), namely (1) photochemical transformation, (2) oxidation, (3) reduction, (4) dissolution and precipitation, (5) adsorption and desorption, (6) combustion, (7) biotransformation and biodegradation and (8) abrasion or mechanical erosion.

While these processes have been discussed in the literature with respect to the exposure assessment of nanoproducts in the use phase (Nowack et al. 2012), most of the processes are also applicable in the context of waste management. In most modern waste management systems, where waste is collected and treated relatively quickly, photochemical transformation can be considered the least relevant of these transformation processes. However, the potential for photochemical transformation should not be ignored, as the exposure of nanowaste, for example to sunlight, may still be possible (e.g. during collection, storage prior to treatment and open-dump landfilling). In a landfill, ENMs release into infiltrating water may be determined by processes such as reduction, dissolution/precipitation and adsorption/desorption. During waste incineration, the main process is combustion, indicating that ENMs may end up in flue gas or the solid residues from the incinerator. Biodegradation and biotransformation are relevant processes in biological waste treatment (e.g. anaerobic digestion and composting). For example, ENMs may be emitted during waste collection because of abrasion during mechanical compaction (Roes et al. 2012). During recycling processes, material shredding and sorting may involve mechanical erosion.

Actual release into the environment may therefore not only occur ‘within’ the actual waste treatment facility, but also in several cases could take place during the management of residual streams coming from waste facilities, for example, the treatment of flue gas cleaning residues, the management of sludge from treating landfill leachate and the application of digestate on land.

### Step 5: Assessment of potential exposure

With the identification of possible environmental compartments into which ENMs are emitted from solid waste, the final step in the exposure assessment framework is to determine the potential magnitude, frequency and duration of exposure. Most waste treatment facilities and processes are operated continuously and for long periods (upto decades in some cases), meaning that potential exposure is rather constant and long term. The magnitude of the exposure depends then on the concentration and amount of the emission and the geography of the population/system exposed.

A quantitative assessment of potential exposure is currently rather difficult—if possible at all—as data are scarce, knowledge about release mechanisms limited and most suitable metrics for exposure quantification and reporting subject to intense discussion (see “Discussion” section of this paper). A qualitative approach is thus hereby adopted, where potential exposure is identified as low, medium and high, based on a qualitative evaluation of the abovementioned factors. The outcomes of the assessment may be used for identifying hotspots and subsequently planning direct sampling campaigns and experimental activities aiming at increasing data availability. However, when the amount and quality of data increase, this assessment can be replaced by quantitative evaluations.

### Application of the environmental exposure assessment framework to three EOL nanowaste examples

**Fig. 3:**
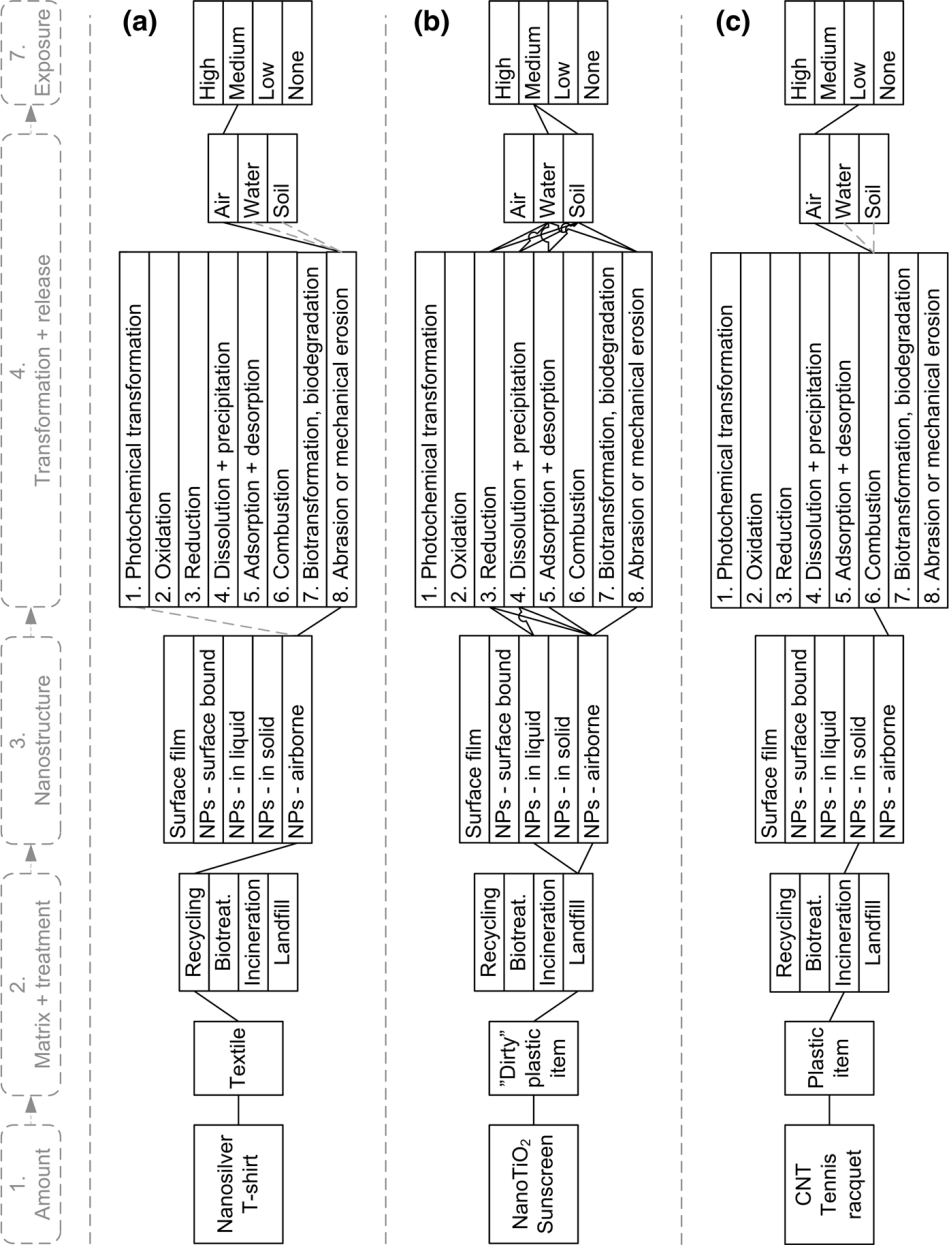
Environmental exposure assessment for the three selected examples: **a** Polyester textile containing nanosilver, **b** Sunscreen lotion containing nanoTiO_2_ and **c** Tennis racquet containing CNT. For details regarding step 1, please refer to Table 3. *Dotted lines* indicate negligible (e.g. photochemical transformation of textiles during recycling) or indirect (e.g. ENMs released into the air or deposited on soil and into water) processes

Three products were selected from Table 2 to test the applicability of the suggested framework for different materials, matrixes, based on data available for 2011. For the individual products, steps 1–5 of the exposure assessment framework presented above were followed. The outcomes of these assessments are presented in the following sections and summarised in Fig. 3 which displays how the framework is applied in practice.

#### Nanosilver in polyester textile

The first example of applying the proposed exposure assessment framework involves nanosilver used in textiles. The outcome of the frameworks is illustrated in Fig. 3a. Nanosilver has strong biocidal properties (Bystrzejewska-Piotrowska et al. 2009) and is used in different ‘odour-free’ clothing products (e.g. T-shirts, socks, etc.), to prevent the formation and subsequent emission of undesirable odours (Emam et al. 2013). Textiles are possibly one of the most important sources of nanosilver in the environment (Lorenz et al. 2012).

##### Step 1: Quantification

The lifespan of textiles is variable and depends very much on individual habits. For example, published lifecycle analysis studies report the lifespan of a textile (a T-shirt is used here as a proxy) ranging from 50 washes (Laursen et al. 2007; Steinberger et al. 2009), to 75 washes (ISR 2009) and upto 100 washes (Walser et al. 2011). Converting the number of washes into a number of years is troublesome, as the conversion factors depend on the frequency a single user washes the cloth. Considering an average 8–16 washes per year (Markus et al. 2013 reported ten washes per year), we assumed that the lifespan of clothing items is on average six years, meaning that production figures for 2005 are needed to estimate the amounts of EOL nanosilver textiles generated in 2011.

The global production (*x*_*t* − *rt*_) of polyester in 2005 was 26.3 × 10^6^ Mg, estimated based on 24.5 × 10^6^ Mg produced in 2004 and an annual growth of 7.2 % (Aizenshtein 2006). Assuming a market penetration (*F*_pen_) of nanosilver textiles of 0.1 % for 2005 (Conde 2009), the global production of nanosilver textiles in 2005 was in the order of 26.3 × 10^3^ Mg, which is in the same order of magnitude as estimated by Lorenz et al. (2012). Very few data are available regarding the loss of textile material during washing and usage. However, an estimation of 10 % loss (i.e. *F*_eol_ = 0.9) was reported by Meyer et al. (2009) and Gottschalk et al. (2010). The amount (*X*_*t*_) of nanosilver textiles in 2011 is thus estimated at 23.7 × 10^3^ Mg (see Table 3 for an overview of the estimated amount of nanowaste in relation to the selected products).

**Table 3:** Generation of nanowaste in 2011 for the selected products: Nanosilver textiles, TiO_2_ sunscreen, CNT tennis racquets

	Nanosilver textile		Sunscreen lotion		Tennis racquet	
*x*_*t* − *rt*_	26.3 × 10^6^ Mg	Aizenshtein (2006)	71.5 × 10^3^ Mg	Calculated	1,650 Mg	Compositesworld (2008)
rt	6 years	Estimated	3 years	Estimated	2–6 years	Estimated
*F*_pen_	0.001	Conde (2009)	>0.1	Boxall et al. (2007)	0.2–0.5	Estimated
*F*_eol_	0.9	Meyer et al. (2009)	0.1–0.2	Estimated	0.95–1	Gottschalk et al. (2010); Franco et al. (2007);
						Meyer et al. (2009)
*X*_*t*_	23.7 × 10^3^ Mg		715–1,430 Mg		313–825 Mg	
*C*_NM_	100 mg/kg	Mueller and Nowack (2008)	10 %	Gottschalk et al. (2010)	1.49 g/kg	Nanoledge (2008)
	240 mg/kg	Walser et al. (2011)	2 %	Mueller and Nowack (2008)		
			5 %	Boxall et al. (2007)		
*F*_NM_	0.55–0.99	Gottschalk et al. (2010)	1		1	Franco et al. (2007); Meyer et al. (2009)
	0.33	Walser et al. (2011)				
NM_t_	0.78–5.6 Mg		14.3–143 Mg		0.47–1.23 Mg	

The concentration (*C*_NM_) of nanosilver in polyester fibres is in the range of 100 g Mg^−1^ (Mueller and Nowack 2008) to 238.5 g Mg^−1^ (Walser et al. 2011), meaning that globally about 2.6–6.3 Mg of nanosilver is used in textile manufacturing in association with polyester. Similar values are estimated using a different approach. Mueller and Nowack (2008) estimated the production of nanosilver in the order of 500 Mg year^−1^ in 2008, meaning that estimated production for 2005 could be in the order of 100–200 Mg year^−1^. Assuming that 10 % of total nanosilver was used in the textile sector (Mueller and Nowack 2008), this means that about 10–20 Mg year^−1^ of nanosilver was used in textile production (i.e. 13–63 % of the nanosilver used in the textile sector was used in association with polyester), which is in line with the previous estimation. Conversely, Keller et al. (2013) estimated that 380–420 Mg year^−1^ of ENMs was used in the textile sector in 2011. Rather than being in disagreement, such estimation should be interpreted as a consequence of the significant growth in the use of ENMs in different applications. In addition to the loss of matrix material, 1–45 % (Geranio et al. 2009; Meyer et al. 2009; Gottschalk et al. 2010) to 67 % (Walser et al. 2011) of the total nanosilver content in textile materials is released throughout the textile’s use phase, mainly during washing, meaning that only a fraction (*F*_NM_) of the initial nanosilver reaches solid waste streams. The amount (NM_*t*_) of nanosilver particles in textile waste in 2011 was thus estimated at 0.78–5.6 Mg.

##### Step 2: Matrix + treatment

In Switzerland, nanosilver textiles are disposed of in the same way as conventional textiles—5 % recycling and 95 % incineration (Walser et al. 2011). A similar situation is likely to occur in countries with a similar waste management system, such as Nordic countries (Arvidsson et al. 2012). In this illustrative example, we focus on nanosilver textile being recycled into, for example, thermal/acoustic insulation material, similarly to what is described in Valverde et al. (2012).

##### Step 3: Nanostructure

Nanosilver is either ‘embedded in’ or ‘coated onto’, for example, the polyester and/or cotton fibres used for the textiles (Walser et al. 2011).

##### Step 4: Transformation + release

During the mechanical shredding process (Fig. 3), at least part of surface-bound ENMs is liberated and released into the air. The recycling facility may be equipped with fume hoods, ventilation and air cleaning systems, but a share of the ENMs may still be released into the environment, as filtration systems are not fully effective in dealing with ENMs (Ling et al. 2012).

##### Step 5: Exposure assessment

The waste management option for textiles was identified as recycling (Step 2), during which the ENM is either embedded or coated in the structure (Step 3), and an environmental exposure assessment has to be related to airborne particles and secondarily through water and soil after ENM deposition (Step 4). Recycling facilities are probably located in populated areas, meaning that both humans and ecosystems may be exposed. As mentioned previously, large amounts of nano-enabled textiles (Step 1) containing significant quantities of ENMs are already on the market, and significant growth is expected in the near future. Ventilation/filtration systems with a significant removal potential may be often installed in recycling facilities. A ‘medium’ level of potential exposure is thus qualitatively associated with nanosilver textiles (Fig. 3a).

#### Nano-scale titaniumdioxide in sunscreen lotion

The second example of applying the proposed exposure assessment framework is nano-scale titaniumdioxide (NanoTiO_2_) used in sunscreen lotion. The outcome of the frameworks is illustrated in Fig. 3b. NanoTiO_2_ particles are blended in sunscreen lotions because of their capacity to absorb and reflect UV light. Sunscreens containing these ENMs are already widespread on the global market; for example, more than 300 registered sunscreen products containing nanoTiO2 are available in Australia (Mueller and Nowack 2008).

##### Step 1: Quantification

The lifespan of sunscreen products is estimated by taking into account that sunscreen lotions have a shelf life of two to two-and-a-half years and, once purchased, are normally used within one season (an average bottle is 200 g, Mueller and Nowack 2008, which is enough for four full-body applications). We thus assume a lifespan of 3 years (same as the expiry date), meaning that EOL sunscreens in 2011 refer to items produced in 2008. Sunscreen lotion containing nanoTiO_2_ is typically contained in plastic flacons, and as the lotion tends to stick to the container, some lotion may not be used and still be present in the flacons at the EOL. These bottles may therefore be disposed together with ‘dirty’ plastic in the residual waste fraction.

The global market for suncare products in 2008 had a volume of 547 × 10^6^ units, 65.4 % of which were sun protection items (Datamonitor 2010). Considering a market penetration (*F*_pen_) of nanoTiO_2_-containing sun lotions of more than 10 % (Boxall et al. 2007) and a mean weight of cosmetics being 200 g (Mueller and Nowack 2008), and also assuming that 10–20 % of the lotion is not used (*F*_eol_) and is still inside the container when thrown away, it is estimated that some 715–1,430 Mg of sunscreen containing nanoTiO_2_ was likely to be contained in the waste stream in 2011 (Table 3). The remaining 80–90 % is applied to the skin and subsequently ends up in environmental compartments, for example, water, where it is no longer collectable and hence not considered waste.

The concentration (*C*_NM_) of TiO_2_ in sunscreen products is in the range of 2–10 % (Mueller and Nowack 2008; Boxall et al. 2007; Gottschalk et al. 2010), meaning that globally 14.3–143 Mg of nanoTiO_2_ was used in sunscreens in 2008 (Table 3). In the same year, the global production of nanoTiO_2_ was in the order of 5,000 Mg year^−1^ (Mueller and Nowack 2008), 0.3–3 % of which was thus used in sunscreens.

##### Step 2: Matrix + treatment

Most of the ENMs blended in cosmetic products are likely to be washed off during showering and will eventually end up in wastewater (Meyer et al. 2009; Keller et al. 2013). The container and the leftover residue, however, will be collected as solid waste. In this example, EOL sunscreen is assumed to be land filled and includes both the plastic bottle and the leftover lotion.

##### Step 3: Nanostructure

In sunscreen lotion, TiO_2_ is suspended in a liquid media. Some ENMs, however, may bind to the surface of the plastic flacon containing the lotion.

##### Step 4: Transformation + release

In the landfill, the flacon may undergo several geochemical processes under the action of infiltrating water (dissolution/precipitation), pH changes (oxidation, reductions) and surrounding media and materials (oxidation, reduction, adsorption/desorption), as shown in Fig. 3. The ENMs will be released mostly into underground water bodies through the leachate, though depending on their mobility, they may also bind to soil.

##### Step 5: Exposure

The waste management option for sunscreen lotion was assumed to be land filling (Step 2). The ENM is suspended in a liquid media (Step 3), so an exposure assessment thus has to be related to the release of ENMs into soil and water. Landfills can be placed in both populated and sparsely populated areas, where significant exposure can be expected for the natural ecosystem, while human exposure is indirect through water and food consumption. Although metal oxides are normally not very mobile, their release from landfill sites may continue for long periods of time. Thus, considering the significant amounts of nano-enabled cosmetics (Step 1) which may be disposed of (see Table 3), potential exposure is defined qualitatively as ‘medium’ (Fig. 3b).

#### Carbon nanotubes in tennis racquets

The third example of applying the proposed exposure assessment framework involves CNTs in tennis racquets. The outcome of the frameworks is illustrated in Fig. 3c.

In tennis racquets, CNT molecules are suspended in the graphite matrix to strengthen and provide rigidity to the structure of the composite.

##### Step 1: Quantification

The lifetime of a tennis racquet is very variable—depending on the frequency and manner of usage—, and no precise data are available. Although a period of between two and six years could be considered a reasonable lifespan for a tennis racquet, a precise number is not necessary for the present modelling. In fact, the market for tennis racquets has been pretty constant for the last decade, and the same can be assumed for the number of racquets being thrown away (i.e. material losses during use phases can reasonably be assumed negligible). In 2006, the amount (*x*_*t* − *rt*_) of carbon fibres utilised for manufacturing tennis racquets was in the order of 1,650 Mg (Compositesworld 2008). Data about the market penetration of tennis racquets using nanotechnology are not available; however, nanotechnologies have been incorporated in tennis racquets since early 2000, and all major producers now offer models containing nanotechnologies at prices which are close to nano-free equipment. It is, thus, reasonable to assume a market penetration (*F*_pen_) value of at least 20 % and upto 50 %. Considering that 95 % (Gottschalk et al. 2010) to 100 % (Meyer et al. 2009; Franco et al. 2007) of the initial material will reach the EOL and be disposed of, it is estimated that 313–825 Mg year^−1^ of tennis racquets with CNT will end up in solid waste streams (Table 3).

The content (*C*_NM_) of CNT in tennis racquets is in the order of 1.49 g/kg (Nanoledge 2008), and because CNT molecules are embedded in a solid graphite matrix, no release is likely to occur during the use phase, and thus, 100 % of the ENM will reach EOL (Franco et al. 2007; Meyer et al. 2009). Consequently, it is estimated that about 0.47–1.23 Mg year^−1^ of CNT will be found in solid waste streams (Table 3).

##### Step 2: Matrix + treatment

A tennis racquet may be disposed of with the residual fraction of household solid waste, or it may in some cases be delivered along with bulky waste at a recycling station. It can be, thus, assumed that the tennis racquet will be incinerated.

##### Step 3: Nanostructure

In tennis racquets, CNTs are bound in the graphite solid matrix, a rather resistant material with a long lifespan.

##### Step 4: Transformation + Release

In an incineration furnace, the adiabatic combustion temperature is normally higher than 1,000 °C, which is the combustion temperature of CNT as reported by Mueller et al. (2013), meaning that both the graphite matrix and the CNTs are oxidised into CO_2_ during the combustion process. Modern waste-to-energy plants must fulfil strict (and stricter) regulations regarding emissions, meaning that a flue gas cleaning system downstream of the combustion chamber (and eventually the boiler, if present) should be installed. State-of-the-art flue gas cleaning systems can be considered efficient in removing most non-combusted CNTs (Köhler et al. 2008), indicating that only minor amounts will be emitted into the air through exhaust gas.

##### Step 5: Exposure

Incineration was identified as the waste management option for tennis racquets (Step 2), in which CNTs are bound in the graphite solid matrix (Step 3). Any exposure assessment has to be related to the release of airborne particles (Step 4). Incineration facilities are typically placed in populated (urban) areas, where mostly humans may be exposed to the released ENMs. However, considering the small amounts of racquets (Step 1) and the (small) magnitude of emissions (Step 4), the potential exposure to CNTs contained in tennis racquets can, thus, be considered ‘low’ (Fig. 3c).

## Discussion

Applying the assessment framework in the previous section illustrated that ENMs in individual waste materials may undergo alternative disposal routes, resulting in different exposure pathways. It also emphasised that an assessment of ENM exposure routes in relation to the waste management phase is complex and should include evaluations of all critical aspects in relation to the ENMs, the matrix materials and the potential transformation processes in the waste system, as even small amounts of nanomaterials may potentially have adverse environmental effects (Baun et al. 2009; Stone et al. 2010). While Table 1 attempts to quantify global amounts of nanowaste, exposure to ENMs is indeed significantly related to local conditions, as the amounts and potential effects of ENMs stemming from waste may be highly variable, depending on the geographical and cultural context as well as the local waste management system (e.g. the sunscreen bottle may undergo incineration in some regions). Considering the rapid increase in nanomaterial application in products, the need for systematically addressing exposure through the waste management system is urgent and should be performed at the local scale. The examples presented in the previous section illustrate clearly that the estimation of nanowaste amounts is possible, at least for certain nanoproducts. However, while these estimates are associated with considerable uncertainties, due to existing data quality, a range of critical aspects can be identified and provide a basis for the focus of future research. These aspects are discussed in the following sections.

### Waste and nanowaste treatment processing

Technological solutions limiting the environmental exposure of ENMs, for example, ventilation and air filtration, are currently available (Lore et al. 2010; Ling et al. 2012; Walser et al. 2011) and allow for removal efficiencies reaching upto 100 % (Liu et al. 2011). Today, however, such technologies are often not applied in general waste management practices, possibly because ENMs in waste are not yet recognised as a key issue. It should be noted that in contrast to Keller et al. (2013), who denied that recycling of ENM-enabled products is currently taking place, we can assume that a number of products being sent for recycling already contain ENMs. As for a range of compounds (e.g. mineral oils in paper, brominated compounds in plastic), the presence of the ENMs may also significantly lower the quality of recycled materials, thereby also lowering the overall recycling potential of a waste stream (if nanowaste is not collected separately). In fact, the presence of unwanted compounds may affect the basic properties (e.g. mechanical) of a material, thus, creating a secondary material of a lower quality and which is thus suitable for fewer applications. In this respect, ENMs can be considered as waste contaminants. The recycling of ENMs as such (i.e. not the matrix) may also be feasible, as reported by Deep et al. (2011) for Zn–MnO_2_ alkaline batteries and Schauerman et al. (2012) for SWCNT anodes from Li-ion batteries. Implementation of specific return systems for individual nanoproducts may, therefore, be a feasible approach for preventing such ENMs from being mixed and disposed of together with the remaining municipal solid waste (SRU 2011). However, at the consumer level, such systems may be applicable only for easily identifiable nanoproducts.

Few studies have focused on the fate of ENMs during the incineration of nanowaste (e.g. Roes et al. 2012; Walser et al. 2011). However, combustion processes involving solid waste are complex, due to the heterogeneity of waste and the effects of process conditions, while flue gas cleaning technologies need to be investigated more thoroughly with respect to the wide variety of ENM types. Subsequent release from incineration residues, for example, by leaching from bottom ashes, should be quantified.

Regarding landfilling of nanowaste, research should focus on how physicochemical and hydraulic conditions in the landfill may affect both the matrix material and the transformation of the ENMs themselves. For example, Nguyen et al. (2011) showed that the degradation of epoxy (potentially containing ENMs) in a landfill was linear over time; however, this may not be the case for all materials. Existing waste research in relation to landfills should be combined with knowledge about the specific properties of ENMs. With regards to their toxicity, Bolyard et al. (2013) indicated that ENMs have no effect on biological activity taking place in leachate; however, such findings need to be confirmed for more types of ENMs and leachate conditions. With respect to final mobility, the release of ENMs into the leachate (and further interaction with other leachate contents) should be addressed further, for example, by examining releases in particulate form or as soluble ions (Liu et al. 2011). In a study focusing on landfill leachate containing high concentrations of humic acids, Lozano and Berge (2012) showed that ENMs can be rather stable and mobile; however, significantly more research is needed to provide a more general overview of the fate of ENMs in landfills (e.g. Asmatulu et al. 2012).

### Characterisation of nanowaste—issues with analytical methods

Characterising material fractions and determining the physicochemical properties of substances in solid waste are basic requirements in decision-making and strategy development in relation to waste management systems. However, as previously indicated, this type of characterisation of nanowaste will be very difficult in practice (von der Kammer et al. 2012). Nanowaste sampling requires the sorting of nanowaste from any remaining waste. This may be a difficult task, as nanowaste itself cannot necessarily be identified (although this may be possible for certain types of EOL nanoproducts, e.g. sunscreen bottles). The physicochemical characterisation of nanowaste includes quantifying ENM concentrations, which cannot be performed using the methods commonly employed for waste analysis (Health Council of the Netherlands 2011; Nowack et al. 2012). This indicates a need for the development of a suitable metrology for nanowaste characterisation, as also highlighted by Musee (2011b). The inherent heterogeneity of solid waste induces a range of significant challenges in relation to nanowaste characterisation:

- ENMs may be lost during solid waste sample preparation, because size reduction operations such as grinding could change the structure of the host material matrix and thereby unintentionally release ENMs during waste sample handling.
- The organic–inorganic nature of some ENMs implies that several analytical methods should be involved.
- Typical methods for determining waste material chemistry are based on the extraction/digestion of the solid matrix and determining elemental composition based on traditional wet chemistry, i.e. the total content of Zn, Ti, etc. However, with this approach, information about types of ENMs or agglomerate/aggregate size, etc. is not provided.
- Available methods used to observe ENMs (e.g. SEM or TEM) could provide information on their type and structure; however, quantitative data in mixed solid materials cannot be obtained from these methods (Markus et al. 2013).
- In many cases, ENM concentrations may be below the detection limit of currently available instruments (Markus et al. 2013).

### Regulation and reporting of nanowaste

When it comes to waste and ENMs, three kinds of potential limitations can be identified in existing regulatory frameworks. First, limitations are related to the definitions of what qualifies nanowaste as a ‘substance’, ‘waste’, ‘hazardous waste’, etc. Second, limitations are linked to the requirements triggered by threshold values not tailored to the nanoscale but which are based instead on bulk material (see e.g. REACH). Third, limitations are related to a lack of metrological tools, (eco) toxicological data and occupational and environmental exposure limits (Hansen and Baun 2012).

As indicated in the previous section, detection and quantification approaches for ENMs in waste are still unresolved, which prevent exposure assessments from delivering the quantitative output usually provided to support risk assessment decisions. Nonetheless, if such data could actually be provided, it is an entirely open question as to how limit values related to ENMs in waste should be defined. Specific regulation based on the concentration of individual ENMs or types of ENMs is most likely unrealistic, especially considering the variety of nanoproducts in which different forms of ENMs can be applied. Metrics addressing relevant ENM properties may be more appropriate for regulatory purposes, for example, as applied to dioxins, furans and PCBs which are expressed as toxic equivalents. The development of such metrics would relate naturally to current research on exposure and effect assessment, especially in relation to human inhalation studies and ecosystem toxicity.

The approaches for reporting nanowaste are identified herein as a crucial input into an exposure assessment scheme. Provided the appropriate quantification methods exist, reporting could be addressed from two perspectives:

- Reporting of the weight of the nanowaste, i.e. also including matrix materials
- Reporting of the amount of ENMs contained in the nanowaste.

Both approaches may be equally relevant, as waste managers are concerned mainly with the waste amounts to be treated, while regulators may be more interested in information about the content and flows of ENMs. While no simple reporting solution necessarily exists, the two aspects of reporting also relate to the definition of nanowaste. Based on the definition presented in this paper, nanowaste should be collected or collectable. When this is the case, the bulk weight of the materials (i.e. the nanowaste) is also known and quantifiable. Reporting on specific ENMs is related to the definition of limit values, while a lack of (eco) toxicological data presently hampers the assessment of whether some forms of nanowaste meet hazard criteria as defined under the Council Directive 1991/689. Meeting these criteria would result in more severe obligations being applied, including the setting of limit and emission values for hazardous substances in waste and requirements for carrying out different forms of recovery (Council Directive 1991/689; Franco et al. 2007).

## Conclusion

The definition of nanowaste has been improved, in order to better reflect the characteristics of modern waste management and to provide improved guidance in relation to the special management of nanowaste, as well as a regulatory definition of limit values and data reporting. On the basis of this definition, a five-step framework for the systematic assessment of potential exposure to nanomaterials in the environment was proposed and discussed: (1) the quantification of nanowaste, (2) the evaluation of matrix properties and nanowaste treatment, (3) the evaluation of the physicochemical properties of the nanostructure, (4) the evaluation of transformation processes and the release of ENMs and (5) the assessment of potential exposure. The framework was applied to three selected nanoproducts (polyester textiles, sunscreen lotion and tennis racquets), indicating that considerable amounts of these nanoproducts entered the waste management system in 2011 (globally 23.7 × 10^3^ Mg of polyester textiles, 715–1,430 Mg of sunscreen lotion and 313–825 Mg tennis racquets). Based on potential waste management practices and exposure routes, this could result in 0.8–5.6 Mg of nanosilver, 14–143 Mg nanoTiO_2_ and 0.5–1.2 Mg CNTs being released annually into the environment on a global scale. Based on the assessment framework, potential environmental exposure from solid waste related to the three nanoproducts was identified as: medium (polyester textiles), medium (sunscreen lotion) and low (tennis racquets). The main challenges in relation to further research within nanomaterials and waste were identified as (1) transformation of nanomaterials within waste treatment technologies, (2) release mechanisms under conditions relevant for waste disposal, (3) exposure assessment performed at the local level within a precise context, (4) the characterisation of nanowaste and the development of appropriate analytical methods and (5) a definition of appropriate regulatory limit values and nanowaste data reporting.

## Electronic supplementary material

Below is the link to the electronic supplementary material.

Supplementary material 1 (DOCX 15 kb)
