# Supplementary material for: Environmental exposure assessment framework for nanoparticles in solid waste
Source: J Nanopart Res. 2014 May 14;16(6):2394. doi: 10.1007/s11051-014-2394-2 (PMC4053593; doi:10.1007/s11051-014-2394-2)
Supplement: Supplementary file 1 — Supplementary material 1 (DOCX 15 kb) [file 11051_2014_2394_MOESM1_ESM.docx]

**Definitions of “pollution” and “waste”**

A definition of “pollution” is provided in Article 2 of COUNCIL DIRECTIVE 96/61/EC:

“*‘pollution’ shall mean the direct or indirect introduction as a result of human activity, of substances, vibrations, heat or noise into the air, water or land which may be harmful to human health or the quality of the environment, result in damage to material property, or impair or interfere with amenities and other legitimate uses of the environment*”.

Relevant definitions concerning “waste” are provided in Article 3 of DIRECTIVE 2008/98/EC:

“*‘waste’ means any substance or object which the holder discards or intends or is required to discard*”

“*‘hazardous waste’ means waste which displays one or more of the hazardous properties listed in Annex III*”

Properties of waste which render it *hazardous*:

H 1 ‘Explosive’

H 2 ‘Oxidizing’

H 3-A ‘Highly flammable’

H 3-B ‘Flammable’

H 4 ‘Irritant’

H 5 ‘Harmful’

H 6 ‘Toxic’

H 7 ‘Carcinogenic’

H 8 ‘Corrosive’

H 9 ‘Infectious’

H 10 ‘Toxic for reproduction’

H 11 ‘Mutagenic’

H 12 Waste which releases toxic or very toxic gases in contact with water, air or an acid.

H 13 (*) ‘Sensitizing’

H 14 ‘Ecotoxic’

H 15 Waste capable by any means, after disposal, of yielding another substance
